# Research Integrity and Research Ethics in Professional Codes of Ethics: Survey of Terminology Used by Professional Organizations across Research Disciplines

**DOI:** 10.1371/journal.pone.0133662

**Published:** 2015-07-20

**Authors:** Dubravka Komić, Stjepan Ljudevit Marušić, Ana Marušić

**Affiliations:** 1 Medical student, University of Split, School of Medicine, Split, Croatia; 2 Rogor, Zagreb, Croatia; 3 Department of Research in Biomedicine and Health, University of Split, School of Medicine, Split, Croatia; Tilburg University, NETHERLANDS

## Abstract

Professional codes of ethics are social contracts among members of a professional group, which aim to instigate, encourage and nurture ethical behaviour and prevent professional misconduct, including research and publication. Despite the existence of codes of ethics, research misconduct remains a serious problem. A survey of codes of ethics from 795 professional organizations from the Illinois Institute of Technology’s Codes of Ethics Collection showed that 182 of them (23%) used research integrity and research ethics terminology in their codes, with differences across disciplines: while the terminology was common in professional organizations in social sciences (82%), mental health (71%), sciences (61%), other organizations had no statements (construction trades, fraternal social organizations, real estate) or a few of them (management, media, engineering). A subsample of 158 professional organizations we judged to be directly involved in research significantly more often had statements on research integrity/ethics terminology than the whole sample: an average of 10.4% of organizations with a statement (95% CI = 10.4-23-5%) on any of the 27 research integrity/ethics terms compared to 3.3% (95% CI = 2.1–4.6%), respectively (P<0.001). Overall, 62% of all statements addressing research integrity/ethics concepts used prescriptive language in describing the standard of practice. Professional organizations should define research integrity and research ethics issues in their ethics codes and collaborate within and across disciplines to adequately address responsible conduct of research and meet contemporary needs of their communities.

## Introduction

There are many definitions of professional codes of ethics and their functions [1] but they can generally be described as formal documents sending a message to the professional community about moral standards guiding professional behaviour. These moral standards also address research and publication activities in most professional societies across disciplines [1]. Judging from the increasing evidence for the seriousness of research misconduct at the global level [2–4], ethics codes have failed miserably in preventing research misconduct, as the practical definition of an ineffective code is that it “has failed to prevent illegal or unethical behaviour… that was prohibited in the code” [5].

Just as there are many definitions of professional codes of ethics, there are also many definitions of research integrity and misconduct, which vary a lot according to the legislative definitions in different countries [4]. In general, research integrity can be defined as “research behaviour viewed from the perspective of professional standards” and is different from research ethics, which is “research behaviour viewed from the perspective of moral principles” [6]. Research integrity (RI) is a part of responsible conduct of research–the ideal behaviour in research, contrasted by deliberate misconduct on the other side of the behavioural spectrum, which includes fabrication, falsification and plagiarism (FFP) as the worst behaviour [6]. In the middle of this behavioural spectrum are the so-called questionable research practices (QRP), which “violate traditional values or commonly accepted practices, from initial project design through to publication and peer review” [6]. Questionable research practices include inaccuracy, misrepresentation and bias in research and publishing [4,6].

Despite the importance of research integrity, it is not clear how professions define and communicate this concept to their membership. There is a wealth of research into codes of ethics, particularly in business [1,7] but little data on how current concepts of research integrity and research misconduct are addressed in the codes. A study of 90 codes from 61 scientific professional organizations funded by the National Science Foundation in the USA in 1998 demonstrated that only 39% had general statements on the need to give proper credit in publications and only 17% provided a definition of authorship [8]. The codes used mostly normative, prescriptive language to describe the “minimum levels of appropriate behaviour” [8]. A comparison of codes/policies from peer-reviewed journals and professional organizations, showed that 53% of the journals and only 11% of professional codes had authorship definitions [9]. Professional codes of ethics used a prescriptive language more often than journals (75% vs 18%) in defining authorship [9]. In a qualitative study of 46 scientific organization codes of ethics [10], the codes included issues such as honesty in conducting and reporting research; fairness and integrity in authorship; appropriate use of public funds; sharing, preservation and dissemination of research results; and responsibility for the integrity of the published record (for organizations with strong publishing activity).

This article attempts to provide the basic landscape for research integrity in professional codes of ethics across different disciplines. We took advantage of the existence of a large online collection of professional codes of ethics, created and maintained by The Center for the Study of Ethics in the Professions (CSEP) from the Illinois Institute of Technology [11]. The Codes of Ethics Collection database was started in 1996, when it was developed through a grant from the US National Science Foundation [12]. The codes are classified into 28 different categories of professional organizations and have been contributed by academic organizations, businesses, industry associations, fraternal organizations, government organizations, non-profit organizations and professional associations.

## Methods

We searched all codes from the Codes of Ethics Collection database for the corpus of research integrity/ethics terms generated from literature and discussion with experts, and counted these statements. We developed the list of research integrity/ethics terms from the definitions provided in the article by N. Steneck from the Office of Research Integrity of the US Department for Health and Human Services in 2006 [6]. We first created a list of 23 terms, which were piloted with a group of 5 researchers in the area of research integrity and ethics (listed in the Acknowledgment section: 1 editor of a medical journal and researcher in publishing integrity and ethics, 3 researchers in moral reasoning and research integrity and ethics, and 1 editor and researcher, former Chair of the Committee on Publication Ethics, COPE). After consultations, some of the terms were rephrases and 3 new terms were added. The list was then discussed with the participants of the 3^rd^ World Congress on Research Integrity (May 2013), where we presented our preliminary analysis [13]; the discussion resulted in addition of one more term to designate conflict of interest (‘dual relationship’). The final search set included 27 RI concepts (in alphabetical order): 1) Author/Authorship, 2) Bias (including bias due to conflict of interest), 3) Competing interest, 4) Conflict of interest, 5) Contributor/Contribution, 6) Credit, 7) Dishonesty, 8) Dual interest/relationship, 9) Ethics, 10) Fabrication, 11) Falsification, 12) Fraud/Fraudulent, 13) Honesty, 14) Inaccuracy, 15) Integrity, 16) Malpractice, 17) Manipulation, 18) Misconduct, 19) Misrepresentation, 20) Plagiarism, 21) Questionable publication practices (QPP)–duplicate publication, 22) QPP–redundant publication, 23) QPP–repetitive publication, 24) QPP–salami publication, 25) QPP–secondary publication, 26) Questionable research practices, 27) Responsible conduct of research.

For analysis, we classified the terms into three groups, which are usually described to span the whole spectrum of research behaviour–from responsible conduct of research (RCR) over questionable research practices (QRP) to research misconduct (FFP–fabrication, falsification, and plagiarism) [6]. “Questionable research practices” is the term used by the Office of Research Integrity in the USA to describe “actions that violate traditional values of the research enterprise and that may be detrimental to the research process”, but are not directly damaging to research as is FFP [6]. The 27 terms identified for this study were arbitrarily divided into these three groups: RCR–‘ethics’, ‘responsible conduct of research’, ‘integrity’, ‘honesty’, ‘authorship’, ‘contributorship’, and ‘credit’; QRP–‘inaccuracy’, ‘misrepresentation’, ‘questionable research practices’, ‘bias’, ‘conflict of interest’, ‘competing interest’, ‘dual interest/relationship’, and ‘questionable publication practices (duplicate, redundant, repetitive and salami publications)’; and research misconduct–‘falsification’, ‘fabrication’, plagiarism’, ‘misconduct’, ‘malpractice’, ‘fraud’, ‘manipulation’ and ‘dishonesty’. We deliberately used overlapping terms and synonyms, as well as terminology related to research ethics, in order to increase the sensitivity of the search, so that we could identify all and any ethics statement that would use these terms in the context of research. Where needed, we used variations of the term to increase the sensitivity of the search (e.g. ‘author’ and ‘authorship’; ‘contribution’, ‘contributor’ and ‘contributorship’; and ‘fraud’ and ‘fraudulent’). Only the statements where the search terms were used to address research activity and not only professional duties were included in the analysis. The last search was performed in October 2013. We did not use any time-limits to the search, so different codes of the same organizations were retrieved. For the code documents with the same title but different dates of issue, only the latest version was analyzed. Documents with different titles from the same professional organization were all analyzed, irrespective of the date of issue. Data extraction and analysis was performed by two authors (DK and AM), with high inter-rater agreement (kappa = 0.997, 95% confidence interval (CI) 0.995–0.999). In cases of disagreement, the two raters discussed the discrepancies and made a consensus decision on the inclusion of a statement in the final analysis. We first analyzed the statements mentioning research integrity/ethics terms from all retrieved professional organizations and then performed a separate analysis for research-related professions. Organizations involved in performing or regulating research were identified as those having the term ‘research’ or ‘science’ in their name or the following terms: ‘academy’, ‘alliance’, ‘association’, ‘board’, ‘center/centre’, ‘chamber’, ‘committee’, ‘congress’, ‘council’, ‘federation’, ‘institute’, ‘journal’, ‘society’, or ‘university’.

For statements included in the analysis, we assessed the tone of the language in the statements addressing research integrity/ethics terms, using the method developed by Rose [8]. The language of a statement was categorized as either *aspirational*, when it formulated suggestions for best or desired practices, such as using the words “strive to,” “attempt to,” “endeavour to,” or “seek” or *prescriptive*–*normative*, when the statement defined minimal standards for practice which should not be failed by any researcher. For example, the statement “*I shall strive to avoid scientific and professional misconduct including*, *but not limited to fraud*, *fabrication*, *plagiarism*, *concealment*, *inappropriate omission of information*, *and making false or deceptive statements*.” was considered aspirational, and the statement *“*… *members shall not commit scientific misconduct*, *defined as fabrication*, *falsification*, *or plagiarism*.” was classified as prescriptive. Two authors (SLM and AM) independently coded the language of the retrieved statements. Kappa index for agreement in coding ranged from 0.719 to 1 for individual terms; the median kappa for all coded terms was 0.940 (95% CI = 0.924–1.000). All differences were resolved by discussion and final agreement on the language coding.

The data were presented as frequencies for categorical variables and means or medians with 95% confidence interval (CI) for continuous variables, depending of the normality of data distribution, as tested by D'Agostino-Pearson test (MedCalc statistical software v.13.0.2; Ostend, Belgium). No statistical tests were employed for comparisons among research integrity/ethics terms or organizations since sampling was not performed. Student t-test for independent samples was used to compare the subsample of research-related organizations with the total sample.

## Results

### Prevalence of research integrity/ethics terms in professional codes of ethics

At the time of the search, the database had a collection of electronic formats of ethic codes from 795 professional organizations. Out of those, 182 (23%) organizations had codes with at least one research integrity/ethics term (full database in S1 File, list of organizations in Table A in S2 File). Most of the organizations that addressed any research integrity/ethics term in their codes were national societies or associations (n = 142, 78%), followed by international societies/associations (n = 20, 11%); there were 7 government institutions (4%, all from USA), 5 universities/institutes (3%), 5 business corporations (3%) and 3 journals (2%).

The number of terms (concepts) addressed by an organization ranged from 1 to 20, with a median of 2 (95% CI 2–3). The body with the highest number of research integrity/ethics terms addressed (20 out of 27) was the International Committee of Medical Journal Editors (ICMJE), followed by the National Oceanic and Atmospheric Administration (United States Department of Commerce) which addressed 17 terms, while the Academy for Certification of Vision Rehabilitation and Education Professionals, American Chemical Society, American Sociological Association and United States Fish and Wildlife Service addressed 14 terms each.

Most commonly addressed research integrity/ethics terms (more than 5% of 795 organizations) were ‘inaccuracy’, ‘ethics’, terms related to authorship and credit for research, ‘plagiarism’, ‘conflict of interest’ and ‘integrity’ (Table 1).

**Table 1:** Research integrity/ethics statements in ethics codes of professional organizations (n = 795) in the Codes of Ethics Collection, ranked by the number of organizations with a statement addressing the term.

Research integrity topic	No. organizations with a code (% all organizations)	No. of statements	Statements with prescriptive language (row %)*
Inaccuracy	79 (10)	112	74 (70)
Contributor/Contribution	65 (8)	99	63 (64)
Ethics	63 (8)	113	68 (60)
Plagiarism	59 (7)	74	53 (72)
Credit	56 (7)	81	62 (77)
Author/Authorship	55 (7)	78	49 (63)
Conflict of interest	53 (7)	72	44 (61)
Integrity	48 (6)	82	34 (42)
Bias†	35 (4)	56	25 (45)
Honesty	33 (4)	48	31 (65)
Falsification	32 (4)	34	24 (71)
Fabrication	29 (3)	30	24 (77)
Fraud/Fraudulent	26 (3)	37	19 (51)
Misrepresentation	26 (3)	41	28 (68)
Misconduct	24 (3)	55	29 (53)
Manipulation	11 (1)	16	7
Questionable publication practices (QPP)–duplicate publication	10 (1)	11	8
Dishonesty	6 (1)	7	3
Dual interest/relationship	6 (0.8)	6	5
Competing interest	4 (0.5)	4	1
QPP–redundant publication	4 (0.5)	5	2
Responsible conduct of research	3 (0.4)	3	2
QPP–repetitive publication	2 (0.3)	2	2
QPP–secondary publication	2 (0.3)	3	1
Questionable research practices	1 (0.1)	1	0
Malpractice	0	0	0
QPP–salami publication	0	0	0

*Percentages were not calculated for groups with n<20.

†These statements included bias due to the conflict of interest.

We separately searched for three terms related to the concept of giving proper credit for research contribution: ‘author(ship)’, ‘contributor(ship)’ and ‘credit’ (Table 1). Whereas ‘authorship’ was defined in a total of 78 statements, 46% of ‘contributor(ship)’ statements (n = 46 of 99) were a part of an authorship definition. ‘Credit for research’ was addressed independently of authorship or contributorship in 42 (52%) of all statements mentioning this concept (n = 81, Table 1).

After deduplication of organizations and statements, terms related to deliberate misconduct, including FFP, were addressed by 78 organizations (10%), with a total of 253 statements. Among these concepts, ‘plagiarism’ was the term addressed by most of the organizations (7%) and in the largest number of statements (Table 1). The so-called Questionable research practices, as defined by the Office of Research Integrity in the USA [6], were addressed by 119 (15%) organizations in 313 statements. Questionable publishing practices [6], such as ‘duplicate’, ‘redundant’ or ‘secondary publication’ were rarely addressed by professional organizations: only 13 (1.6%) organizations addressed any of these concepts, with a total of 19 statements. Whereas no organizations provided any instruction on ‘salami publications’ or ‘salami slicing’ (least-publishable unit or publishing a single study in several partial publication) [6], ‘duplicate publication’ (publishing of the same data more than once without reference to the earlier version) [6] was addressed in 11 statements by 10 (1.3%) organizations (Table 1).

We also analyzed a subset of ethical codes from 158 professional organizations we judged to be directly involved in research (Table 2) (list in Table B in S2 File). These organizations significantly more often had statements on research integrity/ethics terms than the whole sample of professional organizations: average of 10.4% (95% CI = 10.4-23-5%) on any of the 27 concepts compared to 3.3% of organizations with a statement (95% CI = 2.1–4.6%), respectively (t_df = 52_ = 4.186, P<0.001). The ranking of most frequently used terms was similar to that observed in the total sample.

**Table 2:** Research integrity (RI) statements in ethics codes of research-related professional organizations (n = 158) in the Codes of Ethics Collection, ranked by the number of organizations with a statement addressing the term.*

Research integrity topic	No. organizations with a code (% all organizations)	No. of statements	Language with prescriptive language (row %)†
Inaccuracy	62 (39)	88	60 (68)
Contributor/Contribution	61 (39)	96	63 (66)
Ethics	58 (37)	106	64 (60)
Author/Authorship	52 (33)	94	56 (60)
Credit	52 (33)	71	51 (72)
Plagiarism	51 (32)	63	42 (67)
Integrity	42 (27)	74	27 (36)
Conflict of interest	44 (29)	57	32 (56)
Bias‡	29 (18)	51	22 (43)
Honesty	27 (17)	39	24 (62)
Falsification	26 (16)	26	17 (69)
Fabrication	22 (14)	22	16 (73)
Fraud/Fraudulent	22 (14)	30	16 (53)
Misrepresentation	21 (13)	33	23 (70)
Misconduct	20 (13)	41	21 (51)
Manipulation	10 (64)	13	5
Questionable publication practices (QPP)–duplicate publication	8 (5)	9	6
Dual interest/relationship	5 (3)	5	4
Responsible conduct of research	2 (1)	2	0
Dishonesty	3 (2)	3	2
Competing interest	4 (3)	4	1
QPP–redundant publication	4 (3)	5	2
QPP–repetitive publication	2 (1)	2	2
QPP–secondary publication	2 (1)	3	0
Questionable research practices	1 (0.6)	1	0
Malpractice	0	0	0
QPP–salami publication	0	0	0

*Percentages were not calculated for groups with n<20.

†Organizations involved in performing or regulating research were identified as those having the term ‘research’ or ‘science’ in their name or the following terms: ‘academy’, ‘alliance’, ‘association’, ‘board’, ‘center/centre’, ‘chamber’, ‘committee’, ‘congress’, ‘council’, ‘federation’, ‘institute’, ‘journal’, ‘society’, or ‘university’.

‡These statements included bias due to the conflict of interest.

### Language of statements addressing research integrity/ethics terms

We analyzed a total of 1072 statements retrieved by individual term searches, representing 652 unique statements because some individual statements addressed more than one RI concept. Overall, 62% of all statements used prescriptive language in describing the standard of practice. For the analysis of statement language tone for individual research integrity/ethics terms the total set of 1072 statements was used.

Terms addressing responsible conduct of research were mostly described in prescriptive language (309 (61%) of the total of 504 statements) (Table 1). The concept addressed with slightly more statements in aspirational than prescriptive language was ‘integrity’ (58% vs 42%, respectively) (Table 1).

Statements describing research misconduct were also written predominantly in prescriptive language (159 (63%) of 253 statements). Whereas the statements on research fabrication, falsification and plagiarism (FFP) were prescriptive in almost three thirds of the statements, the language of statements describing ‘manipulation’, ‘dishonesty’, ‘fraud’ and ‘misconduct’ was mixed, with equal prevalence of the two language tones or small dominance of aspirational (for ‘dishonesty’ and ‘manipulation’) (Table 1).

Prescriptive language also dominated in the statements addressing the so-called questionable research practices, as 190 (61%) statements out of total 313 used the normative tone. The statements related to research misconduct more often included the description of a procedure to address the breach of integrity (15% of the statement for research misconduct vs 2% for responsible conduct of research concepts and 1% for questionable research practices).

Prescriptive language also dominated in the statements from the subgroup of 158 professional organizations directly related to research (Table 2). There were no differences in the prevalence of prescriptive language among statements for research integrity/ethics concepts: average prevalence of 49.0 (95% CI 37.9-60-1%) for the research professional organizations compared to 53.9% (95% CI 43.8–64.0%) for the total sample (t_df = 52_ = –0.668, P = 0.507).

### RI statements in different professional fields

The above analysis included all statements from individual organizations. However, the results we obtained may not be the reflection of the actual visibility or awareness of these concepts in different research disciplines. The Codes of Ethics Collection database organizes ethics codes into 28 categories, where some organizations are included in more than one category. For example, the Committee on Publication Ethics (COPE) was included in 5 categories: ‘Communications’, ‘Media’, ‘Other Professions’, ‘Science’, and ‘Social Science’s, whereas World Medical Association was included in ‘Health Care’ and in ‘Service Organizations’. Table 3 presents the analysis of research integrity/ethics terms addressed and the language of the statements across different professional disciplines, regardless of their overlap in included organizations, in order to assess the visibility of research integrity/ethics concepts within a discipline rather than in individual professional organizations.

**Table 3:** Research integrity (RI) statements in ethics codes of all professional organizations in 27 categories of the Codes of Ethics Collection, in alphabetical order of organization categories.

Categories of professional organizations (total No. organizations in a category)*	Organizations with a statement (%)	Number of 27 RI concepts addressed (%)	Total number of statements	Statements with prescriptive language (%)†
Agriculture (n = 11)	6 (55)	12 (44)	24	23 (96)
Animal Breeding and Care (n = 18)	7 (39)	16 (59)	38	24 (63)
Architecture, Art and Design (n = 18)	4 (22)	5 (18)	6	6
Business (n = 61)	8 (13)	14 (52)	32	24 (75)
Communications (n = 21)	8 (38)	21 (78)	102	17 (17)
Computer and Information Science (n = 52)	8 (15)	8 (30)	13	11 (85)
Construction Trades (n = 17)	0 (0)	0	0	0
Education and Academia (n = 68)	28 (41)	17 (63)	174	94 (54)
Engineering (n = 48)	4 (8)	9 (33)	20	14
Finance (n = 35)	2 (11)	2 (8)	3	3
Fraternal Social Organizations (n = 5)	0 (0)	0	0	0
Government and Military (n = 106)	9 (8)	18 (67)	90	66 (73)
Health Care (n = 100)	47 (47)	24 (89)	280	162 (58)
Industrial (n = 26)	4 (15)	14 (52)	35	10 (29)
Law and Legal (n = 31)	3 (10)	3 (11)	4	2
Management (n = 22)	1 (5)	15 (56)	20	15
Marketing (n = 12)	2 (17)	4 (15)	10	9
Media (n = 62)	4 (6)	15 (56)	43	4 (9)
Mental Health/Counselling (n = 24)	17 (71)	18 (67)	134	106 (79)
Other Professions (n = 70)	17 (24)	19 (70)	83	34 (41)
Real Estate (n = 6)	0 (0)	0	0	0
Religion (n = 18)	3 (17)	12 (44)	26	24 (92)
Science (n = 75)	46 (61)	23 (85)	478	261 (55)
Service Organizations (n = 22)	10 (45)	17 (63)	39	20 (51)
Social Sciences (n = 17)	14 (82)	19 (70)	133	54 (41)
Sports and Athletics (n = 10)	1 (10)	3 (11)	6	4
Travel and Transportation(n = 14)	2 (14)	10 (37)	13	9
Wildlife and Environmental Stewardship (n = 13)	2 (15)	14 (52)	35	27 (77)

*An organization may be included in more than one professional category in the Collection (795 unique organizations; total of 982 organizations in 28 categories).

†Percentages were not calculated for groups with n<20.

A median of 15% of organizations in any category (95% CI 10–335) had a statement that addressed research integrity/ethics concepts. This prevalence ranged from 0% in categories ‘Construction Trades’, ‘Fraternal Social Organizations’ and ‘Real Estate’ to 82% in ‘Social Sciences’, 71% in ‘Mental Health and Counselling’, and ‘Science’ (Table 3). Most of the organizations addressing research integrity/ethics concepts in their codes belonged to the research-related organizations as defined in our study (Table 4).

**Table 4:** Research integrity (RI) statements in ethics codes for research-related professional organizations in 27 categories of the Codes of Ethics Collection.*

Categories of professional organizations (total No. organizations with ay RI statement in a category)†	Organizations with a statement (% total)	Number of 27 RI concepts addressed (%)	Total number of statements	Statements with prescriptive language (%)‡
Agriculture (n = 6)	5	11 (41)	19	19
Animal Breeding and Care (n = 7)	4	7 (26)	20	12
Architecture, Art and Design (n = 4)	4	5 (20)	5	3
Business (n = 8)	3	11 (41)	17	10
Communications (n = 8)	6	21 (78)	102	17 (17)
Computer and Information Science (n = 8)	6	7 (26)	11	11
Construction Trades (n = 0)	0	0	0	0
Education and Academia (n = 28)	24	16 (59)	160	87 (54)
Engineering (n = 4)	4	9 (33)	20	14
Finance (n = 2)	2	2 (8)	3	3
Fraternal Social Organizations (n = 0)	0	0	0	0
Government and Military (n = 9)	0	0	0	0
Health Care (n = 47)	45	24 (81)	260	144 (55)
Industrial (n = 4)	2	14 (52)	33	13 (39)
Law and Legal (n = 3)	1	3 (12)	2	1
Management (n = 1)	1	15 (56)	20	15
Marketing (n = 2)	2	4 (15)	10	9
Media (n = 6)	3	15 (56)	42	3 (11)
Mental Health/Counselling (n = 17)	15	18 (67)	113	90 (80)
Other Professions (n = 24)	17	19 (70)	83	34 (41)
Real Estate (n = 0)	0	0	0	0
Religion (n = 3)	2	12 (44)	23	22 (96)
Science (n = 46)	46	23 (85)	415	217 (52)
Service Organizations (n = 10)	10	17 (63)	39	20 (51)
Social Sciences (n = 14)	13	19 (70)	131	53 (40)
Sports and Athletics (n = 1)	1	3 (11)	6	4
Travel and Transportation(n = 2)	1	1 (4)	1	1
Wildlife and Environmental Stewardship (n = 2)	0	0	0	0

*Organizations involved in performing or regulating research were identified as those having the term ‘research’ or ‘science’ in their name or the following terms: ‘academy’, ‘alliance’, ‘association’, ‘board’, ‘center/centre’, ‘chamber’, ‘committee’, ‘congress’, ‘council’, ‘federation’, ‘institute’, ‘journal’, ‘society’, or ‘university’.

†An organization may be included in more than one professional category in the Collection (795 unique organizations; total of 982 organizations in 28 categories).

‡Percentages were not calculated for groups with n<20.

The average number of research integrity/ethics concepts addressed by a professional discipline was 11.8 (95% CI for the mean 8.9–14.8). No research integrity topics were addressed by organizations in the categories of ‘Construction Trades’ (n = 17 organizations), ‘Fraternal Social Organizations’ (n = 5) and ‘Real Estate’ (n = 6). Organizations in categories ‘Health Care’ addressed 24 (96%), and those in ‘Science’ addressed 24 (89%) out of 27 concepts. The median number of statements per professional discipline was 29 (95% CI 13–42), ranging from 3 for the ‘Finance’ to 478 for the ‘Science’ category. Prescriptive language in the statements predominated across disciplines, with the average percentage of 58.5% (95% CI 47.0%-70.0%).

Although the size of the category, expressed as the number of organizations having a code addressing research integrity/ethics concepts, positively correlated with the number of statements identified for each category (Table 3), there were categories with an exceptionally large number of statements, such as ‘Sciences’, where 46 organizations had 478 statements. The ‘Health Care’ category had 47 organizations, with 280 statements.

In relation to individual concepts, none of the professional disciplines addressed all concepts. The number of organizations addressing an individual research integrity/ethics concept ranged from 2 to 23 (median 17, 95% CI 7–18). RI topics most commonly addressed were: ‘inaccuracy’ (n = 23 professional disciplines), ‘credit’ (n = 21), ‘integrity’ (n = 21), ‘plagiarism’ (n = 19), author (n = 19), ‘contributor’ (n = 19), ‘honesty’ (n = 18), ‘conflict of interest’ (n = 18), ‘falsification’ (n = 18), ‘fabrication’ (n = 18) and ‘misconduct’ (n = 17). ‘malpractice’ and ‘salami publications’ were not addressed by organizations in any of the professional disciplines. The concepts addressed by the fewest organization categories were ‘repetitive publication’ (n = 3, categories ‘Health Care’, ‘Science’, ‘Service Organizations’), ‘secondary publication’ (n = 3, categories ‘Communication’, ‘Health Care’ and ‘Science’) and ‘questionable research practices’ (n = 2, categories ‘Science’ and ‘Education and Academia’).

The subsample of professional organizations directly related to research did not differ from the total sample (Table 4), in the average number of concepts addressed by organizations (average of 12.6%, 95% CI 9.4–15.8%; range 1 to 415), average number of statements per professional organization of 20.0%, 13.1–64.8%), prevalence of statements with prescriptive language (average of 58%, 95% CI 47.0–70.0%), number of statements per professional categories, and number of organizations addressing individual concepts (average of 11.1 organizations, 95% CI 8.8–13.5%).

## Discussion

Our survey demonstrated that the important terms (concepts) concerning the broad field of research integrity and ethics are not in the focus of professional communities, despite high prevalence of research misconduct and violations of responsible conduct of research [2–4]. The fact that only 23% of 795 professional organizations had a code that addressed at least one of the well-known and generally accepted research integrity/ethics terms [6] is not good news for the scientific community. It is also worrying that even those organizations that defined research integrity concepts in their codes only addressed a small number of important terms, from 2 to 3 per organization. In the subsample of professional organizations directly involved in research (n = 158), codes of ethics on average addressed three times more RI terms that the whole sample. In both groups, the language of the statements on research integrity/ethics terms was predominantly prescriptive, setting minimal standards which must not be failed. In this way, a strong message is sent to its members about expected professional behaviour.

Some professional fields, such as ‘Education and Academia’, ‘Health Care, ‘Mental Health/Counselling’, ‘Science’ and ‘Other Professions’ (as classified in the Collection) had the highest number of organizations and the highest number of statements addressing research integrity/ethics concepts per organization. This indicates that some professions, especially those providing care for human individuals or providing teaching services pay special attention to research as an important aspect of their work. The most commonly addressed research integrity/ethics concepts were ethics and authorship/contributorship/credit from the “positive” spectrum of research behaviour, and inaccuracy, plagiarism and conflict of interest among the” negative” spectrum of research behaviour. These concepts are very old, and provide the base for moral judgments in any profession [10]. Newer research integrity/ethics concepts, such as ‘responsible conduct of research’ and ‘questionable research practices’, commonly used in research on research integrity [6], seem not to have found their way into all professions.

A limitation to the study is the fact that the Collection of Codes may not be representative of the research community, which is most acutely concerned with research integrity. However, 475 out of 795 analyzed organizations (60%) had the term ‘association’, ‘federation’, ‘society’, ‘academy’, ‘college’, ‘university’, ‘congress’ or ‘council’, or ‘science’ in their title. Even in this subsample of professional organizations that should address research and publishing activities the prevalence of research integrity statements was only 38%. When we analyzed a subsample of organizations that may be directly related to research (judging from their names) and that addressed research integrity/ethics concepts in their codes, we found a greater number of concepts addressed than in the whole sample. Also, organizations from this subsample were responsible for 82% of the statements addressing research integrity/ethics concepts in the whole sample. However, we would argue that this distinction between research and non-research professional organizations is artificial. Although research is not explicitly mentioned in most of the definitions of professional codes of ethics [1], it is implicit that a profession should be engaged in collecting evidence and using it for its further development. This is reflected in the definition of profession by Cogan [14] as a “vocation whose practice is founded upon an understanding of the theoretical structure of some department of learning or science, and upon abilities accompanying such understanding.” This is illustrated by the fact that the organizations from the ‘Government and Military’ and ‘Wildlife and Environmental Stewardship’ categories did not include research-related organizations, but still addressed important research integrity/ethics concepts (9 ‘Government and Military’ organizations addressed 18 concepts in 90 statements and 2 Wildlife and Environmental Stewardship’ organizations addressed 14 concepts in 35 statements). Furthermore, some of the disciplines traditionally considered as research-oriented, had a small prevalence of organizations with a code addressing research integrity/ethics concepts, such as 8% for ‘Engineering’.

Another limitation of the study is the fact that we analyzed only the codes available online. It is possible that professional organizations have relevant guidelines in a printed form or on a web-site different from the one provided in the Collection. For a few that were not transcribed into the database, such as the one from the International Committee of Medical Journal Editors, we analyzed the content of the website provided in the link. We did not attempt to retrieve the codes that were available only in a printed version. Our intention was to investigate the codes available in the public domain because one of the important aspects of a profession is to provide service, i.e. have a public purpose [1]. Our search strategy was designed to be sensitive, so that the statements with any term related to research integrity/ethics could be identified. This resulted in terminological overlaps and synonyms, which were all included in the analysis. We also did not perform a qualitative analysis of the content of the statements, so it is possible that brief and vague statements that included more than one research integrity/ethics term would contribute more to the frequency analysis in this study than a long and detailed statement with a single term. The search strategy also did not have a time limit, which introduced a bias, as our intention was to provide a time–independent landscape of research integrity/ethics concepts in professional organizations. Furthermore, the Collection is dominated by organizations from the USA and thus biased towards scientific communities in developed, high-income countries. In view of this fact, the survey findings are even more worrying because evidence shows that research misconduct is at least as prevalent in low–and middle–income countries as it is in high–income countries [4], and in some aspects of research misbehaviour, such as authorship [3] or plagiarism [15], it can even be a greater problem. Finally, the categorization of codes and language of the statement was a subjective and arbitrary process and thus prone to bias; however, the agreement in coding between two independent reviewers was high, indicating consistency in the applied methodology, and the terminology was developed in collaboration with experts in research integrity research and based on commonly used terms in this community (such as current World Conferences on Research Integrity and past RI research on research integrity conferences [6]).

The findings of our study should inform professional organizations to revise and update their codes to include current concepts in research integrity and ethics. Such a change will probably not guarantee the change in research behaviour, as the current evidence base for the effectiveness of codes of ethics in changing behaviour is controversial [7]. However, as professional organizations are moral agents in a self-organized community [16], they have an influence on the moral judgments of that same community and the public in general through the profession’s engagement in providing a service to the public. Furthermore, as research integrity is behaviour in research related to professional standards and not necessarily only moral standards [6], it would be easier for the professional organizations and the professional community in general to establish and implement such standard than to ensure strict adherence to moral rules. Most of the professional organizations that had addressed research integrity/ethics concepts in their codes used the prescriptive language in the statements, establishing a norm for a professional behaviour. Such language tone sends a clear signal about the minimal standards for professional practice in responsible conduct of research, i.e. working rules for everyday professional research activities [8,10]. Such language may not be applicable to concepts that are more related to research ethics than to integrity. Research on the codes of ethics in business [17] showed that the use of language may greatly influence the perception of a code among its users. For example, overuse of grammatical structures such as relational clauses, the passive, nominalisation, grammatical metaphor and modality may communicate an authoritarian message and sense of over-obligation, which establishes a feeling of powerlessness and the inability for open decision making for the individual [17]. This may deter a professional from using professional codes of ethics, as was shown in a national survey of physicians in the USA, where only one in four practicing physician acknowledged a strong influence of the traditional (Hippocratic) oath or other professional codes in their practice, relying rather on their own personal moral sense [18].

Professional organizations need also to address how their professional standards in research are presented to the public. The quality of a code of ethics depends on its pubic availability, involvement of the governing structures, readability and tone, non-retaliation and reporting, commitment and values, risk topics, comprehension aids and presentation and style [19]. The quality of the code has to be integrated in a complex process of code development and implementation, where the success at the level of stakeholders in a profession and the society as a whole are determined by factors both internal and external to the profession. Professional communities should also collaborate across disciplinary borders and share experiences in defining, preventing and dealing with research integrity and research misconduct. A good example of trans-disciplinary collaboration is a recent exercise from the US Institute of Medicine, which worked on a unified code of ethics for health professionals from 18 different disciplines related to health [20]. Only by taking a serious and conscientious approach to research integrity, professional communities in different disciplines can make their codes of ethics relevant to the changing landscape of science.

## Supporting Information

S1 File Database of ethic codes with statements on research integrity terms analysed in the study. (XLSX)

S2 File List of all professional organizations (Table A) and professional organizations directly involved in research activities (Table B). (DOCX)
