# Supplementary material for: Research Integrity and Research Ethics in Professional Codes of Ethics: Survey of Terminology Used by Professional Organizations across Research Disciplines
Source: PLoS One. 2015 Jul 20;10(7):e0133662. doi: 10.1371/journal.pone.0133662 (PMC4507982; doi:10.1371/journal.pone.0133662)
Supplement: S2 File — (DOCX) [file pone.0133662.s002.docx]

**Table A.** List of all professional organizations with ethics codes that address any aspect of research integrity

| **Code category** | **Professional organizations** |
| --- | --- |
| **Agriculture** | American Association of Agricultural and Biological Engineers |
|  | Crop Science Society of America |
|  | Ecological Society of America |
|  | International Society of Arboriculture |
|  | Society of American Foresters |
|  | United States Government, Department of Agriculture |
| **Animal Breeding and Care** | International Association of Animal Behavior Consultants, Inc. |
|  | International Association of Veterinary Editors |
|  | National Institutes of Health Office of Laboratory Animal Welfare |
|  | Oregon Chapter of the American Fisheries Association |
|  | Swiss Academy of Medical Sciences, Basel ; Swiss Academy of Natural Sciences, Bern |
|  | The Wildlife Society |
|  | U.S Fish and Wildlife Service |
| **Architecture, Art and Design** | American Institute for Conservation of Historic and Artistic Works |
|  | American Institute of Architects |
|  | Architectural Institute of British Columbia |
|  | Royal Institute of British Architects |
| **Business** | Canadian Marketing Association |
|  | Employee Assistance Professionals Association |
|  | Exelon Corporation |
|  | International Chamber of Commerce / European Society for Opinion and Marketing Research |
|  | Journal of International Business Studies |
|  | Market Technicians Association, Inc. |
|  | McGraw-Hill Companies |
|  | Monsanto |
| **Communications** | International Committee of Medical Journal Editors |
|  | American Geophysical Union |
|  | American Society of Business Publication Editors |
|  | American Speech, Language, and Hearing Association |
|  | Committee on Publication Ethics |
|  | International Society for Medical Publication Professionals |
|  | Journal of International Business Studies |
| **Computer and Information Science** | American Health Information Management Association |
|  | American Society for Information Science |
|  | British Computer Society |
|  | Centro de la Informatica, Telematica y medios Afines |
|  | Computer Society of India |
|  | IEEE-CS/ACM Joint Task Force on Software Engineering Ethics and Professional Practices |
|  | International Business Machines (IBM) |
| **Education and Academia** | Academy for Certification of Vision Rehabilitation and Education Professionals |
|  | American Anthropological Association |
|  | American Association of University Professors |
|  | American Educational Research Association |
|  | American Historical Association |
|  | American Mathematical Society |
|  | American Political Science Association |
|  | American Sociological Association |
|  | American Statistical Association |
|  | Archaeological Institute of America |
|  | Association for Education and Rehabilitation of the Blind and Visually Impaired |
|  | Association for Educational Communications and Technology |
|  | Association for Institutional Research |
|  | Association of American Medical Colleges |
|  | Association of College Honor Societies |
|  | Association of Social Science Researchers |
|  | Australian Association of Consulting Archaeologists Inc |
|  | British Library Board |
|  | Campbell University |
|  | Cornell University |
|  | Council for Exceptional Children |
|  | Illinois Institute of Technology Code of Ethics |
|  | NAFSA: Association of International Educators |
|  | National Council on Public History |
|  | Society for Applied Anthropology |
|  | Sociological Society of Aotearoa |
|  | University of Connecticut |
|  | University of Illinois, Champaign/Urbana |
| **Engineering** | American Association of Cost Engineers. |
|  | American Institute of Aeronautics and Astronautics |
|  | Biomedical Engineering Society |
|  | National Society of Professional Engineers |
| **Finance** | American Institute of Certified Planners |
|  | Canadian Society of Technical Analysts |
| **Government and Military** | Illinois Department of Children and Family Services |
|  | National Oceanic and Atmospheric Administration (United States Department of Commerce) |
|  | State of Connecticut |
|  | State of Virginia |
|  | United States Fish and Wildlife Service |
|  | United States Government, Department of Agriculture |
|  | United States Government, Department of Health and Human Services |
|  | United States Government, Department of the Army |
|  | United States Government, National Aeronautics and Space Administration |
| **Health Care** | Academy for Certification of Vision Rehabilitation and Education Professionals |
|  | American Academy of Neuorology |
|  | American Academy of Ophthalmology |
|  | American Academy of Optometry |
|  | American Academy of Orthopaedic Surgeons |
|  | American Alliance for Health, Physical Education, Recreation and Dance |
|  | American Association for Clinical Chemistry |
|  | American Association for Respiratory Care |
|  | American Association of Nurse Anesthetists |
|  | American Board of Industrial Hygiene |
|  | American Chiropractic Board of Sports Physicians |
|  | American College of Emergency Physicians |
|  | American College of Occupational and Environmental Medicine |
|  | American College of Physicians |
|  | American College of Radiology |
|  | American College of Rheumatology |
|  | American Dance Therapy Association |
|  | American Dental Association |
|  | American Dental Hygienists' Association |
|  | American Health Information Management Association |
|  | American Medical Informatics Association |
|  | American Music Therapy Association |
|  | American Nurses Association |
|  | American Occupational Therapy Association |
|  | American Osteopathic Association |
|  | American Physical Therapy Association |
|  | American Society of Exercise Physiologists |
|  | American Speech, Language, and Hearing Association |
|  | Argentine Medical Association |
|  | Association of American Medical Colleges |
|  | Australian Medical Association |
|  | Barcelona Biomedical Research Park |
|  | Canadian Nurses Association |
|  | Commission on Rehabilitation Counselor Certification |
|  | International Association of Forensic Nurses |
|  | International Commission on Occupational Health |
|  | International Committee of Medical Journal Editors |
|  | International Council of Nurses |
|  | International Society for Medical Publication Professionals |
|  | International Society for Neurofeedback and Research |
|  | National Commission for the Protection of Human Subjects of Biomedical and Behavioral Research |
|  | National Society of Genetic Counselors |
|  | Society of Pediatric Pathology |
|  | Swiss Academy of Medical Sciences, Basel ; Swiss Academy of Natural Sciences, Bern |
|  | United States Government, Department of Health and Human Services |
|  | World Health Organization |
|  | World Medical Association |
| **Industrial** | American Association of Petroleum Geologists |
|  | American Board of Industrial Hygiene |
|  | American Chemical Society |
|  | Dow Corning Corporation |
| **Law and Legal** | California Association of Criminalists |
|  | IMS Expert Services |
|  | State of Delaware |
| **Management** | Academy Of Management |
| **Marketing** | American Association for Public Opinion Research |
|  | Canadian Marketing Association |
| **Media** | American Society of Business Publication Editors |
|  | Committee on Publication Ethics |
|  | International Society for Medical Publication Professionals |
|  | Kansas City Star |
| **Mental Health/Counseling** | Addiction Counselor Certification Board of Oregon |
|  | American Association for Intellectual and Developmental Disabilities |
|  | American Association for Marriage and Family Therapy |
|  | American Association of Christian Counselors |
|  | American Association of Pastoral Counselors |
|  | American Counseling Association |
|  | American Mental Health Counselors Association (AMHCA) |
|  | American Music Therapy Association |
|  | American Psychoanalytic Association |
|  | Australian Psychological Society |
|  | B.C. Association of Clinical Counsellors |
|  | British Association for Counselling and Psychotherapy |
|  | British Psychological Society |
|  | Canadian Psychological Association |
|  | Christian Association for Psychological Studies |
|  | National Board for Certified Counselors |
| **Other Professions** | American Alliance for Health, Physical Education, Recreation and Dance |
|  | American Association for Public Opinion Research |
|  | American College of Vedic Astrology |
|  | American Culinary Federation |
|  | American Evaluation Association |
|  | American Institute of Certified Planners |
|  | Association of Art Museum Curators |
|  | Association of Professional Genealogists |
|  | Canadian Museum Association |
|  | Committee on Publication Ethics |
|  | Council of American Survey Research Organizations |
|  | Institute of Food Science and Technology |
|  | Institute of Food Technologists |
|  | International Council of Museums |
|  | National Council of Geocosmic Research |
|  | Science Museum of Minnesota |
|  | World Archaeological Congress |
| **Religion** | American Association of Christian Counselors |
|  | American Association of Pastoral Counselors |
|  | Spiritual Care Collaborative |
| **Science** | Acoustical Society of America |
|  | Alaska Native Science Commission |
|  | American Alliance for Health, Physical Education, Recreation and Dance |
|  | American Anthropological Association |
|  | American Association for Clinical Chemistry |
|  | American Association for the Advancement of Science |
|  | American Association of Petroleum Geologists |
|  | American Chemical Society |
|  | American Council of Independent Laboratories |
|  | American Geophysical Union |
|  | American Institute of Aeronautics and Astronautics |
|  | American Institute of Biological Sciences |
|  | American Institute of Physics |
|  | American Mathematical Society |
|  | American Meteorological Society |
|  | American Physical Society |
|  | American Psychological Association |
|  | American Society for Biochemistry and Molecular Biology |
|  | American Society for Microbiology |
|  | American Sociological Association |
|  | American Statistical Association |
|  | Archaeological Institute of America |
|  | Association of Social Anthropologists of the UK and Commonwealth |
|  | Australian Academy of Science |
|  | Barcelona Biomedical Research Park |
|  | Biomedical Engineering Society |
|  | Committee on Publication Ethics |
|  | Ecological Society of America |
|  | Estuarine Research Federation |
|  | Human Factors and Ergonomics Society |
|  | Institute of Food Science and Technology |
|  | International Association of Veterinary Editors |
|  | International Committee of Medical Journal Editors |
|  | International Society of Ethnobiology |
|  | International Sociological Association |
|  | National Oceanic and Atmospheric Administration (United States Department of Commerce) |
|  | National Society for the Practice of Anthropology |
|  | Protein Science |
|  | Register of Professional Archaeologists |
|  | Royal Society of Chemistry |
|  | Royal Society of New Zealand |
|  | Society of Wetland Scientists |
|  | United States Government, Department of Agriculture |
|  | United States Government, National Aeronautics and Space Administration |
|  | World Archaeological Congress |
| **Service Organizations** | Argentine Medical Association |
|  | Australian Medical Association |
|  | British Association of Social Workers |
|  | Illinois Department of Children and Family Services |
|  | International Council of Nurses |
|  | National Association of Social Workers |
|  | National Organization for Human Services |
|  | World Association of Non-Governmental Organizations |
|  | World Health Organization |
|  | World Medical Association |
| **Social Sciences** | Alaska Native Science Commission |
|  | American Anthropological Association |
|  | American Historical Association |
|  | American Political Science Association |
|  | American Sociological Association |
|  | Association of Professional Genealogists |
|  | Association of Social Anthropologists of Aotearoa/New Zealand |
|  | Association of Social Anthropologists of the UK and Commonwealth |
|  | Association of Social Science Researchers |
|  | Australian Association of Consulting Archaeologists Inc |
|  | Committee on Publication Ethics |
|  | Council of American Survey Research Organizations |
|  | National Society for the Practice of Anthropology |
|  | Register of Professional Archaeologists |
| **Sports and Athletics** | American Alliance for Health, Physical Education, Recreation and Dance |
| **Travel and Transportation** | Air Traffic Control Association |
|  | United States Government, National Aeronautics and Space Administration |
| **Wildlife and Environmental Stewardship** | The Wildlife Society |
|  | United States Fish and Wildlife Service |

**Supplementary table 2.** List of research professional organizations with ethics codes that address any aspect of research integrity

| **Code category** | **Organization** |
| --- | --- |
| **Agriculture** | American Association of Agricultural and Biological Engineers |
|  | Crop Science Society of America |
|  | Ecological Society of America |
|  | International Society of Arboriculture |
|  | Society of American Foresters |
| **Animal Breeding and Care** | International Association of Animal Behavior Consultants, Inc. |
|  | International Association of Veterinary Editors |
|  | National Institutes of Health Office of Laboratory Animal Welfare |
|  | Swiss Academy of Medical Sciences, Basel ; Swiss Academy of Natural Sciences, Bern |
| **Architecture, Art and Design** | American Institute for Conservation of Historic and Artistic Works |
|  | American Institute of Architects |
|  | Architectural Institute of British Columbia |
|  | Royal Institute of British Architects |
| **Business** | International Chamber of Commerce / European Society for Opinion and Marketing Research |
|  | Journal of International Business Studies |
|  | Market Technicians Association, Inc. |
| **Communications** | American Society of Business Publication Editors |
|  | American Speech, Language, and Hearing Association |
|  | Committee on Publication Ethics |
|  | International Committee of Medical Journal Editors |
|  | International Society for Medical Publication Professionals |
|  | Journal of International Business Studies |
| **Computer and Information Science** | American Association for Public Opinion Research |
|  | American Health Information Management Association |
|  | American Society for Information Science |
|  | British Computer Society |
|  | Centro de la Informatica, Telematica y medios Afines |
|  | Computer Society of India |
| **Education and Academia** | American Anthropological Association |
|  | American Association of University Professors |
|  | American Educational Research Association |
|  | American Historical Association |
|  | American Mathematical Society |
|  | American Political Science Association |
|  | American Sociological Association |
|  | American Statistical Association |
|  | Archaeological Institute of America |
|  | Association for Education and Rehabilitation of the Blind and Visually Impaired |
|  | Association for Educational Communications and Technology |
|  | Association for Institutional Research |
|  | Association of College Honor Societies |
|  | Association of Social Science Researchers |
|  | Australian Association of Consulting Archaeologists Inc |
|  | Campbell University |
|  | Cornell University |
|  | Illinois Institute of Technology Code of Ethics |
|  | NAFSA: Association of International Educators |
|  | National Council on Public History |
|  | Society for Applied Anthropology |
|  | Sociological Society of Aotearoa |
|  | University of Connecticut |
|  | University of Illinois, Champaign/Urbana |
| **Engineering** | American Association of Cost Engineers |
|  | American Institute of Aeronautics and Astronautics |
|  | Biomedical Engineering Society |
|  | National Society of Professional Engineers |
| **Finance** | American Institute of Certified Planners |
|  | Canadian Society of Technical Analysts |
| **Health Care** | American Academy of Neurology |
|  | American Academy of Ophthalmology |
|  | American Academy of Optometry |
|  | American Academy of Orthopaedic Surgeons |
|  | American Alliance for Health, Physical Education, Recreation and Dance |
|  | American Association for Clinical Chemistry |
|  | American Association for Respiratory Care |
|  | American Association of Nurse Anesthetists |
|  | American Board of Industrial Hygiene |
|  | American Chiropractic Board of Sports Physicians |
|  | American College of Emergency Physicians |
|  | American College of Occupational and Environmental Medicine |
|  | American College of Physicians |
|  | American College of Radiology |
|  | American College of Rheumatology |
|  | American Dance Therapy Association |
|  | American Dental Association |
|  | American Dental Hygienists' Association |
|  | American Health Information Management Association |
|  | American Medical Informatics Association |
|  | American Music Therapy Association |
|  | American Nurses Association |
|  | American Occupational Therapy Association |
|  | American Osteopathic Association |
|  | American Physical Therapy Association |
|  | American Society of Exercise Physiologists |
|  | American Speech, Language, and Hearing Association |
|  | Argentine Medical Association |
|  | Association of American Medical Colleges |
|  | Australian Medical Association |
|  | Barcelona Biomedical Research Park |
|  | Canadian Nurses Association |
|  | International Association of Forensic Nurses |
|  | International Commission on Occupational Health |
|  | International Committee of Medical Journal Editors |
|  | International Council of Nurses |
|  | International Society for Medical Publication Professionals |
|  | International Society for Neurofeedback and Research |
|  | National Commission for the Protection of Human Subjects of Biomedical and Behavioral Research |
|  | National Society of Genetic Counselors |
|  | Society of Pediatric Pathology |
|  | Swiss Academy of Medical Sciences, Basel ; Swiss Academy of Natural Sciences, Bern |
|  | United States Government, Department of Health and Human Services |
|  | World Health Organization |
|  | World Medical Association |
| **Industrial** | American Association of Petroleum Geologists |
|  | American Chemical Society |
| **Law and Legal** | California Association of Criminalists |
| **Management** | Academy Of Management |
| **Marketing** | American Association for Public Opinion Research |
|  | Canadian Marketing Association |
| **Media** | American Society of Business Publication Editors |
|  | Committee on Publication Ethics |
|  | International Society for Medical Publication Professionals |
| **Mental Health/Counseling** | American Association for Intellectual and Developmental Disabilities |
|  | American Association for Marriage and Family Therapy |
|  | American Association of Christian Counselors |
|  | American Association of Pastoral Counselors |
|  | American Counseling Association |
|  | American Mental Health Counselors Association (AMHCA) |
|  | American Music Therapy Association |
|  | American Psychoanalytic Association |
|  | Australian Psychological Society |
|  | B.C. Association of Clinical Counsellors |
|  | British Association for Counselling and Psychotherapy |
|  | British Psychological Society |
|  | Canadian Psychological Association |
|  | Christian Association for Psychological Studies |
|  | National Board for Certified Counselors |
| **Other Professions** | American Alliance for Health, Physical Education, Recreation and Dance |
|  | American Association for Public Opinion Research |
|  | American College of Vedic Astrology |
|  | American Culinary Federation |
|  | American Evaluation Association |
|  | American Institute of Certified Planners |
|  | Association of Art Museum Curators |
|  | Association of Professional Genealogists |
|  | Canadian Museum Association |
|  | Committee on Publication Ethics |
|  | Council of American Survey Research Organizations |
|  | Institute of Food Science and Technology |
|  | Institute of Food Technologists |
|  | International Council of Museums |
|  | National Council of Geocosmic Research |
|  | Science Museum of Minnesota |
|  | World Archaeological Congress |
| **Religion** | American Association of Christian Counselors |
|  | American Association of Pastoral Counselors |
| **Science** | Acoustical Society of America |
|  | Alaska Native Science Commission |
|  | American Alliance for Health, Physical Education, Recreation and Dance |
|  | American Anthropological Association |
|  | American Association for Clinical Chemistry |
|  | American Association for the Advancement of Science |
|  | American Association of Petroleum Geologists |
|  | American Chemical Society |
|  | American Council of Independent Laboratories |
|  | American Institute of Aeronautics and Astronautics |
|  | American Institute of Biological Sciences |
|  | American Institute of Physics |
|  | American Mathematical Society |
|  | American Meteorological Society |
|  | American Physical Society |
|  | American Psychological Association |
|  | American Society for Biochemistry and Molecular Biology |
|  | American Society for Microbiology |
|  | American Sociological Association |
|  | American Statistical Association |
|  | Archaeological Institute of America |
|  | Association of Social Anthropologists of the UK and Commonwealth |
|  | Australian Academy of Science |
|  | Barcelona Biomedical Research Park |
|  | Biomedical Engineering Society |
|  | Committee on Publication Ethics |
|  | Ecological Society of America |
|  | Estuarine Research Federation |
|  | Human Factors and Ergonomics Society |
|  | Institute of Food Science and Technology |
|  | Institute of Food Technologists |
|  | International Association of Veterinary Editors |
|  | International Committee of Medical Journal Editors |
|  | International Society of Ethnobiology |
|  | International Sociological Association |
|  | National Society for the Practice of Anthropology |
|  | Protein Science |
|  | Royal Society of Chemistry |
|  | Royal Society of New Zealand |
|  | Society of Wetland Scientists |
|  | World Archaeological Congress |
| **Service Organizations** | Argentine Medical Association |
|  | Australian Medical Association |
|  | British Association of Social Workers |
|  | Illinois Department of Children and Family Services |
|  | International Council of Nurses |
|  | National Association of Social Workers |
|  | National Organization for Human Services |
|  | World Association of Non-Governmental Organizations |
|  | World Health Organization |
|  | World Medical Association |
| **Social Sciences** | Alaska Native Science Commission |
|  | American Anthropological Association |
|  | American Historical Association |
|  | American Political Science Association |
|  | American Sociological Association |
|  | Association of Professional Genealogists |
|  | Association of Social Anthropologists of Aotearoa/New Zealand |
|  | Association of Social Anthropologists of the UK and Commonwealth |
|  | Association of Social Science Researchers |
|  | Australian Association of Consulting Archaeologists Inc |
|  | Committee on Publication Ethics |
|  | Council of American Survey Research Organizations |
|  | National Society for the Practice of Anthropology |
| **Sports and Athletics** | American Alliance for Health, Physical Education, Recreation and Dance |
| **Travel and Transportation** | Air Traffic Control Association |

**Table B.** List of research professional organizations with ethics codes that address any aspect of research integrity

| **Code category** | **Organization** |
| --- | --- |
| **Agriculture** | American Association of Agricultural and Biological Engineers |
|  | Crop Science Society of America |
|  | Ecological Society of America |
|  | International Society of Arboriculture |
|  | Society of American Foresters |
| **Animal Breeding and Care** | International Association of Animal Behavior Consultants, Inc. |
|  | International Association of Veterinary Editors |
|  | National Institutes of Health Office of Laboratory Animal Welfare |
|  | Swiss Academy of Medical Sciences, Basel ; Swiss Academy of Natural Sciences, Bern |
| **Architecture, Art and Design** | American Institute for Conservation of Historic and Artistic Works |
|  | American Institute of Architects |
|  | Architectural Institute of British Columbia |
|  | Royal Institute of British Architects |
| **Business** | International Chamber of Commerce / European Society for Opinion and Marketing Research |
|  | Journal of International Business Studies |
|  | Market Technicians Association, Inc. |
| **Communications** | American Society of Business Publication Editors |
|  | American Speech, Language, and Hearing Association |
|  | Committee on Publication Ethics |
|  | International Committee of Medical Journal Editors |
|  | International Society for Medical Publication Professionals |
|  | Journal of International Business Studies |
| **Computer and Information Science** | American Association for Public Opinion Research |
|  | American Health Information Management Association |
|  | American Society for Information Science |
|  | British Computer Society |
|  | Centro de la Informatica, Telematica y medios Afines |
|  | Computer Society of India |
| **Education and Academia** | American Anthropological Association |
|  | American Association of University Professors |
|  | American Educational Research Association |
|  | American Historical Association |
|  | American Mathematical Society |
|  | American Political Science Association |
|  | American Sociological Association |
|  | American Statistical Association |
|  | Archaeological Institute of America |
|  | Association for Education and Rehabilitation of the Blind and Visually Impaired |
|  | Association for Educational Communications and Technology |
|  | Association for Institutional Research |
|  | Association of College Honor Societies |
|  | Association of Social Science Researchers |
|  | Australian Association of Consulting Archaeologists Inc |
|  | Campbell University |
|  | Cornell University |
|  | Illinois Institute of Technology Code of Ethics |
|  | NAFSA: Association of International Educators |
|  | National Council on Public History |
|  | Society for Applied Anthropology |
|  | Sociological Society of Aotearoa |
|  | University of Connecticut |
|  | University of Illinois, Champaign/Urbana |
| **Engineering** | American Association of Cost Engineers |
|  | American Institute of Aeronautics and Astronautics |
|  | Biomedical Engineering Society |
|  | National Society of Professional Engineers |
| **Finance** | American Institute of Certified Planners |
|  | Canadian Society of Technical Analysts |
| **Health Care** | American Academy of Neurology |
|  | American Academy of Ophthalmology |
|  | American Academy of Optometry |
|  | American Academy of Orthopaedic Surgeons |
|  | American Alliance for Health, Physical Education, Recreation and Dance |
|  | American Association for Clinical Chemistry |
|  | American Association for Respiratory Care |
|  | American Association of Nurse Anesthetists |
|  | American Board of Industrial Hygiene |
|  | American Chiropractic Board of Sports Physicians |
|  | American College of Emergency Physicians |
|  | American College of Occupational and Environmental Medicine |
|  | American College of Physicians |
|  | American College of Radiology |
|  | American College of Rheumatology |
|  | American Dance Therapy Association |
|  | American Dental Association |
|  | American Dental Hygienists' Association |
|  | American Health Information Management Association |
|  | American Medical Informatics Association |
|  | American Music Therapy Association |
|  | American Nurses Association |
|  | American Occupational Therapy Association |
|  | American Osteopathic Association |
|  | American Physical Therapy Association |
|  | American Society of Exercise Physiologists |
|  | American Speech, Language, and Hearing Association |
|  | Argentine Medical Association |
|  | Association of American Medical Colleges |
|  | Australian Medical Association |
|  | Barcelona Biomedical Research Park |
|  | Canadian Nurses Association |
|  | International Association of Forensic Nurses |
|  | International Commission on Occupational Health |
|  | International Committee of Medical Journal Editors |
|  | International Council of Nurses |
|  | International Society for Medical Publication Professionals |
|  | International Society for Neurofeedback and Research |
|  | National Commission for the Protection of Human Subjects of Biomedical and Behavioral Research |
|  | National Society of Genetic Counselors |
|  | Society of Pediatric Pathology |
|  | Swiss Academy of Medical Sciences, Basel ; Swiss Academy of Natural Sciences, Bern |
|  | United States Government, Department of Health and Human Services |
|  | World Health Organization |
|  | World Medical Association |
| **Industrial** | American Association of Petroleum Geologists |
|  | American Chemical Society |
| **Law and Legal** | California Association of Criminalists |
| **Management** | Academy Of Management |
| **Marketing** | American Association for Public Opinion Research |
|  | Canadian Marketing Association |
| **Media** | American Society of Business Publication Editors |
|  | Committee on Publication Ethics |
|  | International Society for Medical Publication Professionals |
| **Mental Health/Counseling** | American Association for Intellectual and Developmental Disabilities |
|  | American Association for Marriage and Family Therapy |
|  | American Association of Christian Counselors |
|  | American Association of Pastoral Counselors |
|  | American Counseling Association |
|  | American Mental Health Counselors Association (AMHCA) |
|  | American Music Therapy Association |
|  | American Psychoanalytic Association |
|  | Australian Psychological Society |
|  | B.C. Association of Clinical Counsellors |
|  | British Association for Counselling and Psychotherapy |
|  | British Psychological Society |
|  | Canadian Psychological Association |
|  | Christian Association for Psychological Studies |
|  | National Board for Certified Counselors |
| **Other Professions** | American Alliance for Health, Physical Education, Recreation and Dance |
|  | American Association for Public Opinion Research |
|  | American College of Vedic Astrology |
|  | American Culinary Federation |
|  | American Evaluation Association |
|  | American Institute of Certified Planners |
|  | Association of Art Museum Curators |
|  | Association of Professional Genealogists |
|  | Canadian Museum Association |
|  | Committee on Publication Ethics |
|  | Council of American Survey Research Organizations |
|  | Institute of Food Science and Technology |
|  | Institute of Food Technologists |
|  | International Council of Museums |
|  | National Council of Geocosmic Research |
|  | Science Museum of Minnesota |
|  | World Archaeological Congress |
| **Religion** | American Association of Christian Counselors |
|  | American Association of Pastoral Counselors |
| **Science** | Acoustical Society of America |
|  | Alaska Native Science Commission |
|  | American Alliance for Health, Physical Education, Recreation and Dance |
|  | American Anthropological Association |
|  | American Association for Clinical Chemistry |
|  | American Association for the Advancement of Science |
|  | American Association of Petroleum Geologists |
|  | American Chemical Society |
|  | American Council of Independent Laboratories |
|  | American Institute of Aeronautics and Astronautics |
|  | American Institute of Biological Sciences |
|  | American Institute of Physics |
|  | American Mathematical Society |
|  | American Meteorological Society |
|  | American Physical Society |
|  | American Psychological Association |
|  | American Society for Biochemistry and Molecular Biology |
|  | American Society for Microbiology |
|  | American Sociological Association |
|  | American Statistical Association |
|  | Archaeological Institute of America |
|  | Association of Social Anthropologists of the UK and Commonwealth |
|  | Australian Academy of Science |
|  | Barcelona Biomedical Research Park |
|  | Biomedical Engineering Society |
|  | Committee on Publication Ethics |
|  | Ecological Society of America |
|  | Estuarine Research Federation |
|  | Human Factors and Ergonomics Society |
|  | Institute of Food Science and Technology |
|  | Institute of Food Technologists |
|  | International Association of Veterinary Editors |
|  | International Committee of Medical Journal Editors |
|  | International Society of Ethnobiology |
|  | International Sociological Association |
|  | National Society for the Practice of Anthropology |
|  | Protein Science |
|  | Royal Society of Chemistry |
|  | Royal Society of New Zealand |
|  | Society of Wetland Scientists |
|  | World Archaeological Congress |
| **Service Organizations** | Argentine Medical Association |
|  | Australian Medical Association |
|  | British Association of Social Workers |
|  | Illinois Department of Children and Family Services |
|  | International Council of Nurses |
|  | National Association of Social Workers |
|  | National Organization for Human Services |
|  | World Association of Non-Governmental Organizations |
|  | World Health Organization |
|  | World Medical Association |
| **Social Sciences** | Alaska Native Science Commission |
|  | American Anthropological Association |
|  | American Historical Association |
|  | American Political Science Association |
|  | American Sociological Association |
|  | Association of Professional Genealogists |
|  | Association of Social Anthropologists of Aotearoa/New Zealand |
|  | Association of Social Anthropologists of the UK and Commonwealth |
|  | Association of Social Science Researchers |
|  | Australian Association of Consulting Archaeologists Inc |
|  | Committee on Publication Ethics |
|  | Council of American Survey Research Organizations |
|  | National Society for the Practice of Anthropology |
| **Sports and Athletics** | American Alliance for Health, Physical Education, Recreation and Dance |
| **Travel and Transportation** | Air Traffic Control Association |
